# CAR19 therapy drives expansion of clonal hematopoiesis and associated cytopenias

**DOI:** 10.21203/rs.3.rs-7746241/v1

**Published:** 2025-10-21

**Authors:** Mark P. Hamilton, Nick Phillips, Troy Noordenbos, Jan Boegeholz, Takeshi Sugio, Brian J Sworder, Stefan K. Alig, Zinaida Good, Joseph G Schroers-Martin, John Tamaresis, Mohammad S. Esfahani, Ying Lu, Mari Olsen, Chih Long Liu, Zach Ehlinger, Moksha Desai, Lori Muffly, Robert S. Negrin, Sally Arai, Laura Johnston, Robert Lowsky, Everett Meyer, Andrew Rezvani, Judith Shizuru, Wen-Kai Weng, Parveen Shiraz, Surbhi Sidana, Sushma Bharadwaj, Melody Smith, Saurabh Dahiya, Bita Sahaf, Matthew J. Frank, Crystal L. Mackall, Maximilian Diehn, David M. Kurtz, David B. Miklos, Ash A. Alizadeh

**Affiliations:** 1 MD Anderson Cancer Center, Department of Lymphoma and Myeloma, Division of Cancer Medicine, Houston, TX 77030, USA.; 2 Center for Cancer Cell Therapy, Stanford Cancer Institute, Stanford University School of Medicine, Stanford, CA 94305, USA.; 3 Division of Blood and Marrow Transplantation and Cellular Therapy, Department of Medicine, Stanford University School of Medicine, Stanford, CA 94305, USA.; 4 Division of Oncology, Department of Medicine, Stanford University School of Medicine, Stanford, CA 94305, USA.; 5 Department of Biomedical Data Science, Stanford University School of Medicine, Stanford, CA 94305, USA.; 6 Department of Radiation Oncology, Stanford University School of Medicine, Stanford, CA 94305, USA.; 7 Stanford Cancer Institute, Stanford University School of Medicine, Stanford, CA 94305, USA.

## Abstract

CD19-directed chimeric antigen receptor T-cell therapy (CAR19) improves survival in patients with relapsed/refractory large B-cell lymphoma (rrLBCL) compared to immunochemotherapy with intent for autologous hematopoietic cell transplantation (HCT). However, major toxicities of CAR19 therapy include prolonged cytopenias, infection, and secondary hematologic malignancies. To investigate the mechanisms underlying these toxicities we studied a cohort of lymphoma patients receiving CAR19. CAR19-treated patients exhibited impaired immune reconstitution and increased infection compared to propensity-matched HCT-treated controls. Bone marrow analysis revealed prolonged post-CAR cytopenias is associated with clonal cytopenias of undetermined significance (CCUS) and is characterized by interferon-mediated inflammation. Despite durable lymphoma remissions, clonal hematopoiesis (CH) commonly expanded following CAR19 infusion and was associated with impaired immune reconstitution and the development of treatment related myeloid malignancy (tMN). The molecular composition and clinical outcomes of post-CAR tMN were comparable to those of post-HCT tMN. Single-cell DNA analysis revealed that most post-CAR CH clones harbored a single independent mutation and that CAR integration into T cells with CH mutations may drive persistence. These findings broadly implicate CH mutation burden and CH expansion in the development of post-CAR cytopenias and malignancies as well as mechanistically suggest these expansions occur in a background of marrow inflammation. Together, our results provide insight into the origins of key CAR19-associated toxicities, including infection and tMN.

## Introduction:

CD19-directed chimeric antigen receptor (CAR19) T-cell therapy has revolutionized the treatment of B-cell malignancies by inducing durable remissions and possibly cures in numerous tumor types^1–11^. Additionally, CAR T-cell therapy is likely to move into the non-malignant space after showing remarkable preliminary efficacy in autoimmune disorders^12^. Despite the success of CAR T-cell therapy concern over toxicity remains. This concern is highlighted both by substantial acute CAR toxicity in the form of cytokine release syndrome (CRS) and immune effector cell associated neurotoxicity syndrome (ICANS)^13^ as well as 5-year non-relapse mortality rates that can reach up to 16%^14–17^ principally due to infection and second primary malignancies^16^.

The major cause of non-relapse mortality in CAR treated patients is infection^16^ and second primary malignancy^18^. Infectious risk is likely linked to persistent cytopenias^19^ which has proven a substantial unexpected risk in this treatment. Lymphodepleting chemotherapy necessary for CAR expansion is expected to cause a degree of cytopenias. However, this chemotherapy does not readily account for the persistent cytopenias noted in many post-CAR patients. CAR cytopenias are defined as early (< 30 days post-infusion), prolonged (30–90 days post-infusion), and late (>90 days post-infusion)^20,21^. Post-CAR cytopenias can persist >1 year post infusion^22,23^ and these cytopenias can include prolonged neutropenia as well as lymphopenia in the form of B-cell aplasia presumably caused by on-target and off-tumor CAR T-cell activity as well as loss of CD4+ T-cells^23^. Post-CAR cytopenias have been linked to persistent CAR T-cell populations which can persist up to 10-years post-infusion^24^. Persistent neutropenia has been subsequently linked to interferon gamma (IFNγ) expressing CD8+ clonal T-cell populations in the bone marrow^25^. The major consequence of prolonged cytopenias is risk for infection which typically includes an immediate risk for bacterial infection followed by an ongoing risk for viral infection^19,23,26,27^. Prolonged cytopenias are linked to pre-infusion cytopenias and inflammation. Post-CAR cytopenias are predictable using clinical tools such as the CAR-HEMATOTOX score^28,29^.

Mutation-driven clonal expansion of hematopoietic stem cells is a ubiquitous age-related change in hematopoiesis and is known as clonal hematopoiesis (CH). Patients following chemotherapy often have increased CH, especially with mutations in DNA damage response genes such as *TP53* or *PPM1D*, representing clonal expansion under selective pressure^30–34^. Consistent with this prior chemotherapy exposure, CAR treated patients do have a high incidence of pre-infusion CH which can be present in 34–48% of patients^35,36^. This pre-existing CH can expand post-infusion^37,38^ and CAR treated patients have increased risk for second primary malignancies principally in the myeloid compartment^39–41^. Pre-existing CH has also recently associated with the development of post-CAR T-cell lymphomas^9,42–46^. These findings complement reports of CAR vector integration into *TET2* and *DNMT3A* clones leading to prolonged survival and efficacy of the CAR T cell^9,47–49^. Pre-infusion CH is linked in other settings to the development of treatment related myeloid neoplasm (tMN)^50^.

Understanding post-CAR cytopenias, related infections, and the development of second malignancies is essential to safely treat patients with CAR T-cell therapy. Balanced by the need to treat lymphoma, secondary malignancies are rare^42,44,51^, and infectious complications are manageable. However, the likely increased use of CAR T-cell therapies in non-malignant populations will shift the risk-benefit associated with each toxicity and may require additional screening measures to identify patients with acceptable risk profiles. Further, the long-term effects of associated secondary malignancies may become relevant years after CAR therapy, yet current information on long-term prevalence and risk remains limited. Secondary malignancy is a known risk of anti-lymphoma therapy^52^ and understanding whether CAR T-cell therapy specifically impacts this risk is critical to defining CAR safety profiles.

To better understand the risk for prolonged cytopenias, infections, and secondary hematologic malignancies we comprehensively profiled a large cohort of patients receiving CAR T-cell therapy for non-Hodgkin lymphoma using clinical outcomes analysis, capture based sequencing, flow cytometry, single cell RNA sequencing, and single cell DNA sequencing. Our results highlight a critical link between development of post-CAR cytopenias, secondary treatment related myeloid neoplasms, and the presence of clonal hematopoiesis.

## Methods:

Molecular methods in this paper were performed according to prior work^42,53–57^. For detailed clinical and molecular methods please see **Supplementary Methods**. All statistical tests were two-sided unless otherwise specified. All confidence intervals are 95% confidence intervals unless otherwise specified.

## Results:

### Slower immune reconstitution and higher rates of severe infection after CAR vs HCT

**Figure 1:**
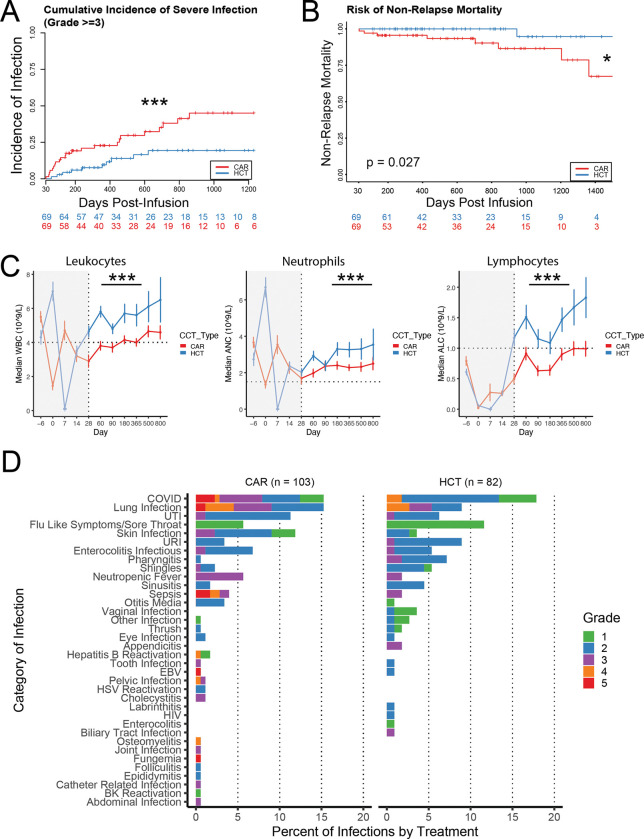
Increased risk of infection and reduced immune reconstitution in CAR- versus HCT-treated patients. **A)** Cumulative incidence of grade ≥3 infections reveal a significantly higher risk among CAR-treated patients compared to HCT-treated controls. **B)** Non-relapse mortality is increased in CAR-treated patients, predominantly due to infectious complications. **C)** Immune reconstitution is delayed after CAR therapy, with reduced leukocyte, neutrophil, and lymphocyte recovery relative to HCT-treated patients. **D)** All infections documented in the CAR vs HCT cohort demonstrating high rates of lung infection and COVID related death in CAR treated patients. CAR, chimeric antigen receptor; HCT, hematopoietic cell transplantation; NRM, non-relapse mortality; ANC, absolute neutrophil count; ALC, absolute lymphocyte count; WBC, white blood cell. * p < 0.05, **p < 0.01, ***p < 0.001.

Comprehensive analysis of non-relapse mortality (NRM) comparing CAR versus HCT is limited. To evaluate the relative risk of cytopenias, infections, and NRM in CAR-treated vs HCT-treated patients we identified 236 CAR treated patients at Stanford relative to 235 HCT treated patients during the same time-period. We 1:1 matched n = 69 non-relapsed patients in each group by age, sex, time from infusion, and prior lines of therapy (**Figure S1A-C, Table S1, File S1–2**). CAR-treated patients experienced a significantly higher cumulative incidence of all-grade infections (p = 0.044; hazard ratio [HR] 1.01–2.37, competing risk regression [CRR], **Figure S1D**) and severe (grade ≥ 3) infections (p = 0.01; HR 1.26–5.41, CRR, Figure 1A). Moreover, there was an increased risk of NRM beyond D+30 in the CAR treated patients principally due to increased death from infection during the study period (0.027, log-rank test, Figure 1B, **Table S2**). Finally, there was significantly delayed immune recovery in terms of white blood cell count (WBC), absolute neutrophil count (ANC), and absolute lymphocyte count (ALC) in the CAR vs HCT group (p<0.001 for each, t-test with Satterthwaite approximation, Figure 1C). There was no difference in hemoglobin recovery between populations and platelet recovery was lower in CAR treated patients on day 28 but recovered (**Figure S1E-F**). Average recovery differences per day as measured by estimated marginal means in 1E9 cells per liter was: WBC −2.08 [confidence interval (CI) −2.65, −1.51], ANC −0.99 [CI −1.39, −0.59], ALC −0.796 [CI −1.02, −0.57].

Analysis of infection types in the larger LBCL cohort (n = 103 CAR, 82 HCT) using common terminology for adverse events (CTCAE) criteria revealed that the most common causes of grade ≥ 3 infections and infection-related death in CAR patients were COVID-19 and lower respiratory infections (Figure 1D). Sepsis, typically of bacterial origin, was the second most common cause of grade 5 infection. There was a similar total percent of COVID and lung infection in the HCT treated group but with lower grades of severity likely due to an improved immune response. The total COVID related mortality in this study was 15.8% for CAR treated patients consistent with reported high COVID related mortality after CAR which is reported as high as 40–50%^15,58,59^. These findings are consistent with recent reports of COVID driving NRM in CAR treated patients in the pandemic era^15,16^. Even when analyzing the competing risk for severe infection without COVID infection the matched population still strongly trended towards increased risk for infection in CAR treated patients (p = 0.075, CRR, HR 0.934 – 4.25).

### CAR T-cell enrichment and persistent CD19 detection in cytopenic CAR bone marrow

Our initial analysis indicated that CAR treated patients demonstrated delayed immune reconstitution relative to HCT treated patients with commensurate increased risk for infection. To investigate the molecular basis of these findings we systematically collected bone marrow biopsies in the post CAR setting. We focused on biopsies after D+85 to consider persistent post-CAR cytopenias (**File S3**). We identified 31 unique post-CAR19 patients with prolonged cytopenias and collected associated molecular sequencing and marrow biopsy data. All patients were in remission from lymphoma at the time of marrow biopsy and remain so for at least three months.

**Figure 2:**
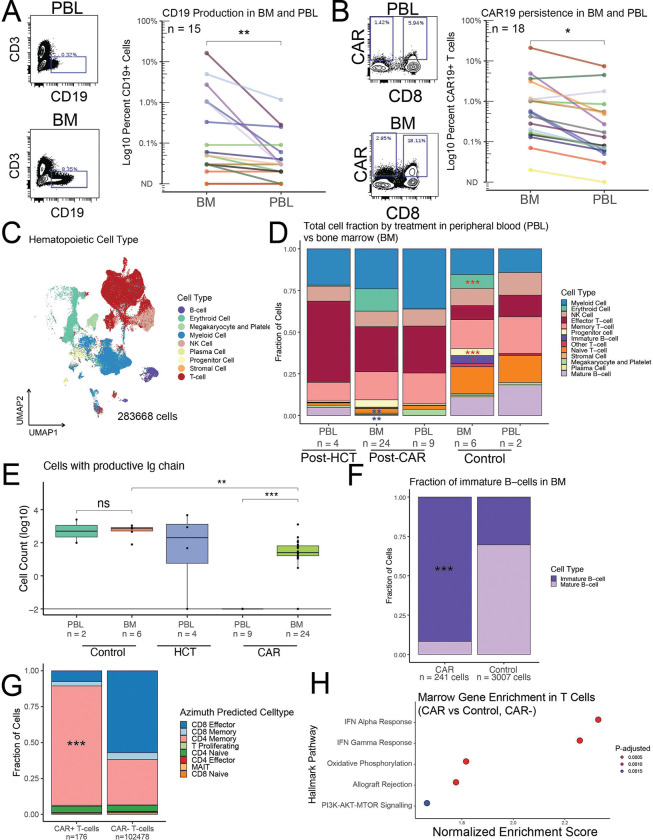
The post CAR marrow environment is associated with persistent B-cell progenitor production, persistent CAR, and elevated inflammatory burden. **A)** Residual production of CD19+ B cells is detected in BM but not PBL of post-CAR cytopenic patients. Representative flow cytometry plots are shown (left), with paired quantification across n = 15 patients (right). **B)** Enrichment of CAR+ T cells within the BM compared to PBL as a percentage of the total CD3+ T-cell population (n = 18). **C)** UMAP clustering of 283,665 single cells from BM and PBL samples across healthy controls, post-HCT patients, and 17 post-CAR patients analyzed by single-cell RNA sequencing (scRNA-seq). **D)** Relative proportions of hematopoietic cell types in PBL and BM by treatment group. Post-CAR BM shows marked T-cell and myeloid skewing with decreased B-cell populations. **E)** Quantification of cells with productive Ig chains in control, post-HCT, and post-CAR samples. Productive Ig rearrangements are observed in post-CAR BM but absent from paired PBL. **F)** Immature B cells (pro-B, pre-B, transitional B cells) dominate the B-cell compartment in post-CAR BM, with near absence of mature B cells. **G)** Residual CAR+ T cells in post-CAR BM are predominantly CD4+, contrasting with the CD8-dominant non-CAR T-cell population. **H)** CAR− CD8+ T cells in post-CAR BM demonstrate enrichment for IFN signaling and inflammatory gene programs. BM, bone marrow; PBL, peripheral blood leukocytes; CAR, chimeric antigen receptor; HCT, hematopoietic cell transplantation; scRNA-seq, single-cell RNA sequencing; UMAP, uniform manifold approximation and projection; Ig, immunoglobulin; IFN, interferon. * p < 0.05, **p < 0.01, ***p < 0.001.

We first conducted molecular profiling on available post-CAR marrow biopsy specimens. Flow cytometry analysis comparing post-CAR marrow aspirates relative to paired peripheral blood leukocytes (PBL) demonstrated CD19 was significantly increased in post-CAR marrow relative to PBLs (p = 0.006, Wilcoxon signed-rank test, Figure 2A, median Hodges-Lehmann fold change 6.67x [CI 1.82–17.5]). Similarly, CAR19+ cells were significantly enriched in post-CAR marrows relative to PBLs (p = 0.019, Wilcoxon signed-rank test, Figure 2B, median Hodges-Lehmann fold change 2.39x [CI 1.87–4.46]). This enrichment was not observed in five control marrow-PBL pairs from healthy donors (**Figure S2A-B**). There was a modest correlation between marrow CAR19 and marrow CD19 levels (R^2^ = 0.52, p = 0.039, Pearson correlation, **Figure S2C**). CD19+CD10+ B-cell progenitor populations were detected post-CAR marrow suggesting ongoing B-cell production with impaired egress into the peripheral circulation (**Figure S2D-E**). These findings suggest that residual CAR19 present in post-CAR BM is sustained by persistent CD19 production from B-cell progenitors. To assess the inflammatory milieu, we performed cytokine profiling on the liquid fraction of marrow aspirates from twelve post-CAR cytopenic marrows relative to four healthy controls. Four proteins (IL-4, PDGF-AA/BB, CCL7, and IL13) were significantly downregulated in the post-CAR cytopenic marrow specimens (t-test with Bonferroni correction). No proteins were significantly upregulated (**Figure S2F**). These results indicated disruption of specific inflammatory and growth factor signaling pathways within the post-CAR marrow microenvironment.

### Definition of post-CAR cytopenic marrow states at single cell resolution

To better characterize the molecular mechanisms underlying post-CAR cytopenias we performed single cell RNA sequencing on 24 post-CAR marrow samples in 19 unique post-CAR patients coupled to five healthy controls (16 post-axi-cel, 1 post-brexu-cel, 2 post-CAR22, **Figures S3A**, **File S4**)^60^. To assess differences between the circulating and marrow population we also included paired PBL samples for n = 8 of these patients as well as four post-HCT controls and two paired healthy controls for a total of 44 unique scRNA datasets accounting for 283668 high quality cells after processing (Figure 2C, **File S4)**. Consistent with successful capture of marrow specimens, marrow samples contained increased proportions of erythroid cells, immature B-cells, and progenitor cells (Figure 2D, red asterisk, beta binomial test with correction)^61^. CAR+ patients globally lacked B-cells and demonstrated reduced naïve T-cell populations relative to control samples, alongside expansion of effector T-cell populations (Figure 2D, purple asterisk, beta binomial test with correction).

Despite the global paucity of B-cells in the post-CAR samples productive immunoglobulin (Ig) chains were detected in post-CAR marrows but not post-CAR PBL indicating ongoing B-cell production in the BM. In contrast, post-HCT samples, which were also exposed to chemotherapy and rituximab, still demonstrated reconstitution of mature B-cells in the peripheral blood at comparable post-infusion timepoints (Figure 2E). Cell type annotation of residual B-cells in the post-CAR marrow revealed marked enrichment of immature B-cell subsets (defined as pre-B, pro-B, and transitional-B) relative to healthy controls (p < 1E-15, Fisher’s exact test, Figure 2F). These findings validate flow cytometry detection of residual CD19+ cells in post-CAR marrow (Figure 2A, **Figure S2D**) and suggests that B-cell progenitors continue to develop in post-CAR marrow but are eliminated by residual CAR T cells. Supporting these findings CD19 expression was reduced in immature B-cells from post-CAR patients relative to controls while CD10 production was intact (**Figure S3B**).

CAR mRNA was detectable in 15/19 (78.9%) post-axi-cel or post-brexu-cel and one of two post-CAR22 patients indicating persistence of the CAR T-cell in post-CAR marrows (**File S4, Figure S3C**). The longest length of CAR detection was more than four years after infusion (**Supplemental File S4**). Despite this persistence CAR+ cells were rare and only CAR19 (axi-cel and brexu-cel) were detected in single cells after data processing (Figure 2E, **Figure S3C**). Lineage assignment using Azimuth^62^ revealed a shift in the CAR+ T-cell population toward a CD4+ memory phenotype, whereas the non-CAR T-cell population was dominated by CD8+ cell types (Figure 2G, **Figure S3C**, p<1E-15, Fisher’s exact test). The non-CAR T-cell population exhibited marked clonality in both the peripheral blood and bone marrow. This clonal T-cell population was almost exclusively defined as CD8 effector cells (**Figure S3D-E**). Similar clonality was seen in the post-HCT peripheral blood (**Figure S3F**). The native (non-CAR) T-cells demonstrated strong IFN signal compared to healthy control T-cells (Figure 2H) indicating there is an infiltrate of inflammatory CD8 effector T-cells in post-CAR marrows that are not present in healthy controls. These findings support prior similar reports of CD8+ inflammatory T-cell populations in post-CAR cytopenic marrows^25^ and indicate that the post-CAR cytopenic marrows are under inflammatory signaling pressure.

### Detection of CH in post-CAR cytopenic marrows

**Figure 3:**
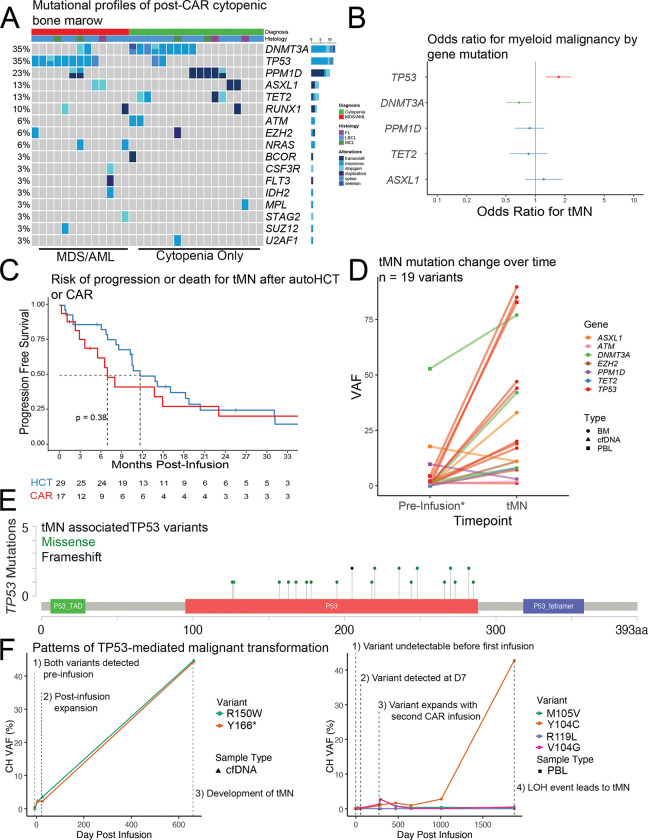
Post-CAR cytopenic marrow is associated with clonal hematopoiesis which drives tMN formation. **A)** Mutational landscape of cytopenic BM samples post-CAR infusion. Most patients harbor canonical CH-associated mutations, including *TP53*, *DNMT3A*, *PPM1D*, and *ASXL1*. **B)** Odds ratios for development of tMN by gene mutation. *TP53* mutations are associated with increased risk of tMN, whereas *DNMT3A* mutations are associated with decreased risk. **C)** Post-infusion tMN (MDS and AML) relapse free survival is not significantly different between post-CAR and post-HCT treated patients. **D)** VAF increase over time for 19 CH-associated mutations that ultimately give rise to tMN. Many mutations are detectable at low VAF in PBL or cfDNA prior to infusion. *Two pre-infusion patient samples are day 7 due to sample availability. **E)** Schematic of tMN-associated *TP53* mutations identified in this cohort, with most alterations mapping to the DNA-binding domain of *TP53*. **F)** Representative patterns of *TP53*-driven malignant transformation. Left: Bi-allelic *TP53* variants detected pre-infusion undergo expansion post-infusion. Right: Single *TP53* mutation detected at Day 7 expands after a second CAR infusion, followed by LOH event causing tMN. BM, bone marrow; PBL, peripheral blood leukocytes; cfDNA, circulating cell-free DNA; VAF, variant allele fraction; tMN, treatment-related myeloid neoplasm; HCT, hematopoietic cell transplantation; CH, clonal hematopoiesis; PFS, progression-free survival; LOH, loss of heterozygosity. * p < 0.05, **p < 0.01, ***p < 0.001.

We next analyzed 31-post-CAR cytopenic patients for whom clinical sequencing data was available. Of these patients 28/31 (90.3%) had associated mutations detected on clinical myeloid capture sequencing panels (Figure 3A, **File S5**) and 13/31 (41.9%) were ultimately diagnosed with MDS or AML based on molecular or histologic features (**Table S3**). Post treatment second primary malignancy remains a major concern after CAR T-cell therapy^63^. Of cytopenic patients without an MDS or AML diagnosis 15/18 (83.3%) met the diagnostic criteria for clonal cytopenias of undetermined significance (CCUS) and 3/18 (16.6%) met the diagnostic criteria for idiopathic cytopenias of undetermined significance (ICUS, n = 1 patient diagnosed with ICUS had a 1% VAF CH clone, two other patients had no clones detected). The most frequently mutated genes were in *DNMT3A* and *TP53* (each 35% of cases), followed by *PPM1D* (23% of cases), *ASXL1* (13% of cases), and *TET2* (13% of cases).

*TP53* gene mutation was significantly associated with development of treatment related myeloid neoplasm (tMN, odds ratio [OR] 1.69 [CI 1.18 – 1.97], logistic regression, Figure 3B) while *DNMT3A* mutations were protective (OR 0.688, [CI 0.506 – 0.933], logistic regression, Figure 3B). In total 9/13 (69.2%) patients with tMN and available molecular analysis harbored *TP53* variants, suggesting *TP53* gene mutations are the predominant driver of malignant myeloid transformation in this population. The majority (6/9) of *TP53-*mutant transformed tMN involved multi-hit (presumed biallelic) *TP53* disruption either at the time of diagnosis or at the time of progression (**File S6**). This included four patients with loss of heterozygosity (LOH) due to deletion of one *TP53* allele and two patients with multiple *TP53* gene mutations.

### Post-CAR treatment related myeloid malignancy is clinically indistinguishable from post-HCT treatment related myeloid malignancy

We next screened 533 post-CAR patients treated for non-Hodgkin Lymphoma (NHL) at Stanford finding 17 total patients with tMN (13 with associated clinical sequencing data are included above). Of these 6/17 (35.3%) had prior autologous transplant and 5/17 (29.4%) were treated with more than one CAR T-cell therapies. In total 11/17 (64.7%) patients with tMN had multiple cellular therapies prior to oncogenic transformation. Among patients with at least 90 days of follow up (n = 448) this association was statistically significant (p < 0.01, logistic regression, **Figure S4A**).

To compare molecular features and clinical outcomes of tMN in the post-CAR setting to patients with similar therapy we screened n = 1007 post-HCT patients treated for non-Hodgkin lymphoma discovering n = 29 patients with treatment related MDS or AML (**Figure S4B**). The post-HCT group had no history of prior cellular therapy, less total lines of therapy, and more cases of T-cell lymphoma in this unmatched analysis (**Table S3**). Despite these differences, post CAR tMN was similar post-auto tMN in this cohort with no difference in age, sex, cytogenetic risk features including complex karyotype, deletion 7, deletion 5, or Revised International Prognostic Scoring System (IPSSR, **Table S3, File S6**)^64^. Similarly, relapse-free survival and overall survival was not significantly different between groups (Figure 3C, **Figure S4C**). Together these data suggest that post-CAR tMN is clinically and molecularly comparable post-HCT tMN.

### Post-CAR tMN typically derives from pre-existing CH

To better understand the origin of post-CAR tMN we next used a capture-based sequencing to discover the temporal origin of tMN associated mutations (**Table S4**). We analyzed peri-infusion (up to day 7) PBLs and cfDNA in 11 patients with post-CAR tMN and traceable mutations. In total we traced 19 tMN-associated mutations to the earliest available timepoint and noted substantial increase in VAF in most documented mutations (median peri-infusion VAF = 0.93%, median VAF after development of tMN = 19%, Figure 3D). Of the 19 tMN-associated mutations 15 were detectable in the peri-infusion samples indicating that the majority of post-CAR tMN traced in this study arose from CH mutations already detectable at the time of infusion.

*TP53* was the predominant gene mutated in tMN cases, frequently affecting the DNA-binding domain of the protein, as expected (Figure 3E). Two dominant patterns of *TP53*-driven tMN were observed: 1) the presence of a likely bi-allelic TP53 gene mutation at infusion (Figure 3F, left panel), and 2) initial expansion of a mono-allelic TP53 gene mutation with a subsequent post-infusion LOH (Figure 3F, right panel; **Figure S4D** demonstrates four additional *TP53* tMN clonal tracings including three LOH and one multi-hit). In one patient who received CAR19 followed by CAR22 the *TP53* gene mutation was first detected at day 7 after the CAR19 infusion. Subsequent CAR22 infusion was followed by clonal expansion and competition of multiple *TP53* gene mutations until a LOH event led to rapid progression into aggressive MDS (Figure 3F, right panel). This case illustrates how sequential cellular therapies may synergize to promote tMN development by accelerating clonal expansions.

### Chemotherapy drives CH formation

**Figure 4:**
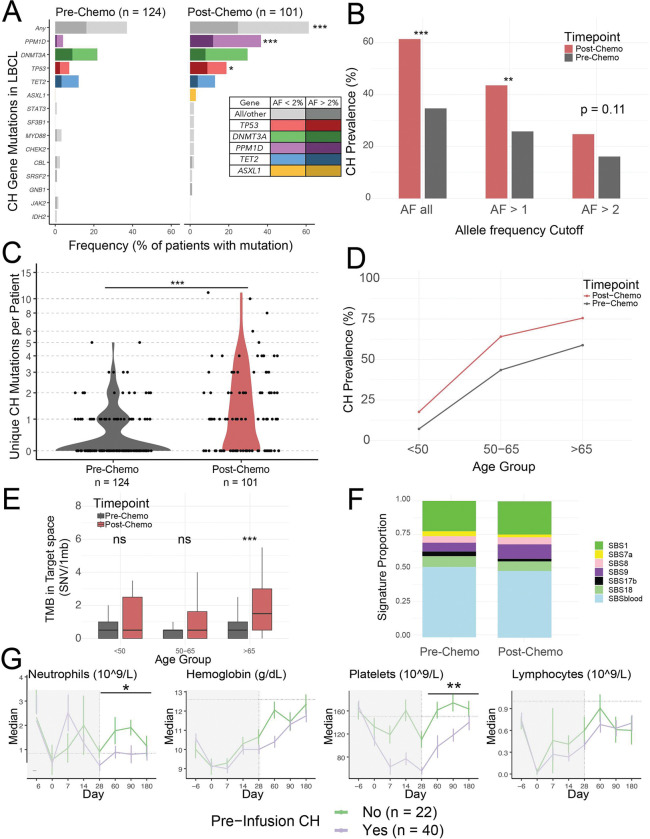
Lymphoma directed chemotherapy leads to increased detectable CH and pre-infusion CH is associated with delayed recovery after CAR. **A)** CH mutations were identified in pre-infusion genomic DNA using our CH panel. Most patients (61%) had detectable CH when including low-VAF variants (<2%). CH was significantly more prevalent in pre-infusion samples than in pre-treatment samples, likely reflecting chemotherapy exposure. **B)** CH prevalence is significantly higher in post-chemotherapy samples across VAF thresholds. **C)** There are additionally more mutations per patient pre-infusion relative to pre-treatment. **D)** The prevalence of CH increases with age in both the pre and post chemotherapy groups. **E)** Older patients have higher TMB after chemotherapy while younger patients do not have higher TMB after chemotherapy. **F)** Mutational signature analysis from ultra-deep sequencing of PBLs reveals dominant contributions from aging-related single base substitution signatures (SBS1 and SBSblood), not lymphoma-associated processes. **G)** The presence of CH prior to CAR T cell infusion was associated with impaired hematopoietic recovery, particularly in neutrophils and platelets when adjusting for age, sex, and CAR-HEMATOTOX score. Includes all CH mutations detected regardless of VAF. CH, clonal hematopoiesis; CAR, chimeric antigen receptor; VAF, variant allele frequency; TMB, total mutational burden. * p < 0.05, **p < 0.01, ***p < 0.001.

Chemotherapy drives CH mutations in canonical DNA damage response (DDR)^30,65^ genes. To assess the impact of chemotherapy on CH development, we compared 124 PBL samples collected from patients with LBCL prior to chemotherapy (“pre-chemo”) with 101 PBL samples obtained after chemotherapy but before second- or third-line CAR infusion (“post-chemo”). This comparison revealed a significant increase in overall CH burden, with enrichment in specific genes such as *TP53* and *PPM1D* after chemotherapy (Figure 4A). This increase was observed across all thresholds of CH mutation calling but was stronger when using lower variant mutation calls (Figure 4B). Using ultra-deep sequencing, 61.3% of patients harbored functional CH mutations in post-chemo PBL samples, compared to 24.7% when applying a VAF threshold of ≥2%, suggesting that the conventional cutoff underestimates the true prevalence of CH. By comparison, in patients sequenced at initial lymphoma diagnosis, CH mutations were detected in 37.1% using any VAF and 16.1% using a ≥2% threshold. Post-chemo patients also harbored more unique CH mutations per individual, with median 1.59 mutations per patient pre-chemo compared to 2.55 mutations post-chemo (Figure 4C).

CH burden increased with age in both the pre-chemo and the post-chemo groups (p < 0.0001 and p = 0.001 respectively, Jonckheere-Terpstra test, Figure 4D). Patients above age 65 who had received prior chemotherapies showed a greater overall mutational burden in circulating cells (Figure 4E). Mutational signature analysis revealed that CH mutations in both pre-chemo and post-chemo cohorts were strongly associated with hematopoietic aging, with over 75% of mutations linked to SBS1 and SBSblood signatures^66^ (73% each, Figure 4F), suggesting that clonal expansion due to age-associated clonal shifts is the most common mechanism driving CH in both cohorts.

Pre- and post-chemo differences in CH burden were similar when controlling for age-related bias by matching 101 pre-chemo and 101 post-chemo samples by age and sequencing depth (**Figure S5A-B**). Both *TP53* and *DNMT3A* have full coverage in our capture panel enabling unbiased mutation analysis. Mutation clustering analysis showed that nearly all *TP53* and *DNMT3A* variants were categorized as potentially deleterious. Consistent with this finding most *TP53* mutations fell within the DNA-binding domain and most *DMNT3A* mutations fell within DNA methylase or PWWP domain (**Figure S5C**). These results indicate that most discovered CH mutations impact protein function.

### CH prior to CAR T-cell infusion is associated with delayed immune recovery

We hypothesized that pre-infusion CH may impact tumor outcomes or immune reconstitution as a measure of *a priori* DNA damage. Focusing on LBCL patients with CH detected within 180 days of CAR T-cell infusion we first analyzed PFS outcomes in patients population finding no difference in PFS consistent with prior reports (**Figure S5D**, **Table S5**)^35,67^. We then assessed immune reconstitution between days 28 and 180 in 62 patients who had not relapsed by day 180. Most patients had detectable pre-infusion CH using ultradeep sequencing methods, and the dataset was enriched for patients with persistent cytopenia. Using linear mixed effects modeling to analyze hematopoietic recovery between day 28 and 180, and adjusting for age, sex, pre-infusion CH, and the CAR- HEMATOTOX^28^ score, we found pre-infusion CH was associated with reduced ANC and platelet recovery (Figure 4G). Average per-day reduction by estimated marginal means in CH+ patients in 1E9 cells per liter were −31.2 [CI −52.82, −9.62] for platelets and −0.65 [CI −1.28, −0.02] for ANC. This impact was most pronounced in the platelets possibly due to growth factor use in patients with neutropenia. These findings align with recent data in multiple myeloma indicating reduced hematopoietic recovery after BCMA CAR T-cell therapy in patients with CH^68^. Delayed immune recovery likely reflects CH as a biomarker of underlying DNA damage, rather than a direct effect of often low-VAF mutations.

The CAR-HEMATOTOX score independently predicted delayed immune recovery in the multivariate model as expected. Pre-infusion CH did not predict subsequent tMN development, including when analysis was restricted to *TP53* mutations (linear regression). Similarly, CH was not associated with ICANS or CRS in a multivariate regression model combined with age and sex. Older age was associated with ICANS development in the same model (p = 0.012, multivariate linear regression).

### Measurement of CH using cfDNA

**Figure 5:**
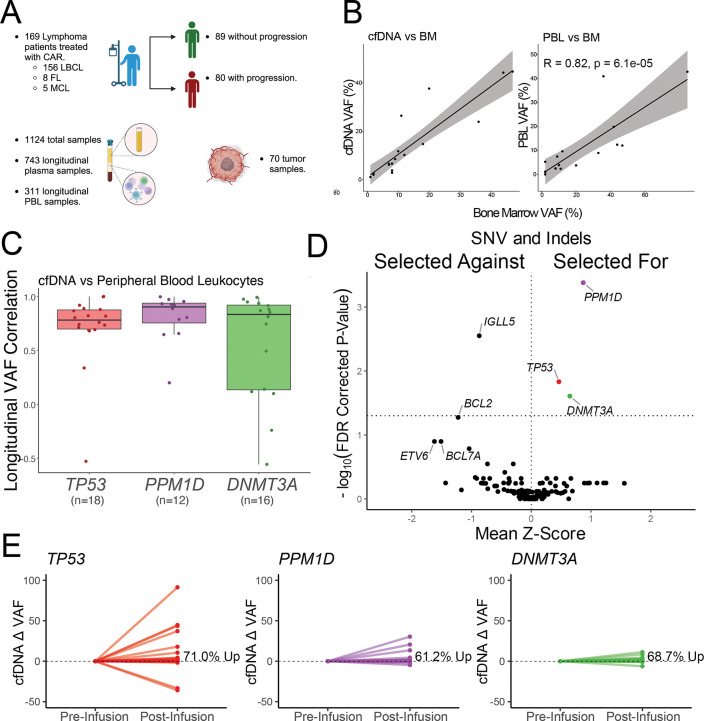
Post-infusion expansion of CH in CAR treated patients. **A)** Schematic of CH mutation profiling in 169 patients with lymphoma treated with CAR19. A total of 1124 samples were analyzed, including longitudinal plasma, peripheral blood leukocytes (PBL), and tumor specimens. **B)** VAFs in cfDNA and PBL correlate strongly with BM VAFs for CH-associated mutations in paired samples. **C)** Longitudinal comparisons of cfDNA and PBL show high concordance in CH mutation dynamics over time, supporting the utility of cfDNA for monitoring CH. **D)** When comparing CH change (pre-infusion vs post-infusion) in patients responding to CAR19 therapy, there is selection for CH mutations in the post-infusion cfDNA indicating expansion of CH as measured by VAF. Includes n = 65 patients, pre-infusion samples are day ≤ 0, post-infusion samples are day ≥ 25. The earliest and latest timepoint is selected for each unique patient. **E)** Gene-specific examples of CH expansion in *TP53*, *PPM1D*, and *DNMT3A*, highlighting the variability in clonal dynamics. LBCL, large B-cell lymphoma; FL, follicular lymphoma; MCL, Mantle cell lymphoma; CH, clonal hematopoiesis; CAR, chimeric antigen receptor; cfDNA, cell-free DNA; PBL, peripheral blood leukocytes; BM, bone marrow; VAF, variant allele frequency.

Our prior findings suggested that post-CAR tMNs arise primarily from pre-existing CH clones. We hypothesized we could use cfDNA to define the clonal dynamics of CH before and after CAR T-cell infusion. We leveraged a pre-existing dataset of 619 tumor, PBL, and cfDNA samples in our CAR cohort^57^ and generated 505 additional cfDNA and PBL samples focusing on longitudinal follow up. The total dataset of capture-based sequencing samples included 1124 unique samples in 169 unique patients (Figure 5A, **File S7**). All samples underwent deep sequencing with a median de-duplicated depth of 2809x allowing analysis of rare CH mutations below the conventional 2% threshold.

To validate cfDNA as a reliable measure of CH, we directly compared VAFs from cfDNA, PBL, and matched clinical bone marrow specimens for known CH mutations and found the cfDNA and PBL VAFs were highly correlated with their clinical bone marrow counterparts (Figure 5B). Because cfDNA can contain tumor-derived circulating tumor DNA (ctDNA) which may confound CH VAF by changing proportionate detection of a given variant we compared likely CH variants in cfDNA and PBL at pre-infusion timepoints, when ctDNA is expected to be elevated, and again observed high correlation (**Figure S6A**). Consistent with a hematopoietic origin, the fragment length of CH mutations did not differ from wildtype fragments (**Figure S6B**). This contrasts ctDNA where mutant fragments have shorter fragment lengths than wildtype fragments^69^. We then assessed whether longitudinal VAF changes in cfDNA and PBL occurred synchronously. Longitudinal analysis of paired PBL and cfDNA samples revealed high temporal concordance (Figure 5C). This correlation held across different PBL sources, including plasma-depleted whole blood (PDWB) and PBMC (**Figure S6C-D)**. This data indicated high linear correlation of detected CH variants between cfDNA, multiple sources of PBL (PDWB and PBMC), and clinical bone marrow specimens validating use of cfDNA to trace CH dynamics over time in longitudinal samples.

### Expansion of clonal hematopoiesis after CAR19 therapy

We hypothesized the CAR therapy may drive general CH expansion. Using methods analogous to our prior work (**Figure S7A**)^57^ but focusing on patients who did not relapse after CAR19 therapy we assessed gene-level variant allele fractions (VAFs) in paired pre- and post-infusion cfDNA to identify evidence of clonal selection (n = 65 patients who had pre-infusion and post-infusion day ≥ 25 cfDNA samples). We found clear evidence for post-infusion gene selection in the CH genes *DNMT3A*, *TP53*, and *PPM1D* (FDR < 0.05, Figure 5D, **Figure S7B**).

When limiting variant calls to likely CH gene mutations, most mutations exhibited increased VAF between the first and last timepoints including 71% of *TP53* mutations, 62.5.6% of *PPM1D* mutations, and 67.3% of *DNMT3A* mutations (Figure 5E). Clonal expansions were often specific to individual gene mutations with intra-patient clonal competition wherein one CH clone expanded dominantly relative to other clones (**Figure S7C**). The doubling time of CH gene mutations in this study was 159 days for PPM1D, 253 days for DNMT3A, 263 days for TP53, and 374 days for all other CH mutations combined. This rate of CH expansion exceeds a recently reported baseline CH doubling time of 7.43 years^70^ by five to ten fold. However, some of this difference may be due to differences in measurement, and most measured changes occurred in the first 90 days after CAR infusion.

Day 7 axi-cel expansion was not significantly associated with increased CH clonal expansion in 44 evaluable patients (**Figure S7D,** p = 0.17 logistic regression). Finally, axi-cel expression at timepoints with tMN was not different than similarly distant timepoints (defined as day ≥ 85). This finding provides no evidence for CAR integration into the studied tMN clones (**Figure S7E**).

### Myeloid-CH and Lymphoid-CH differentially contribute to post-CAR cytopenic states

**Figure 6:**
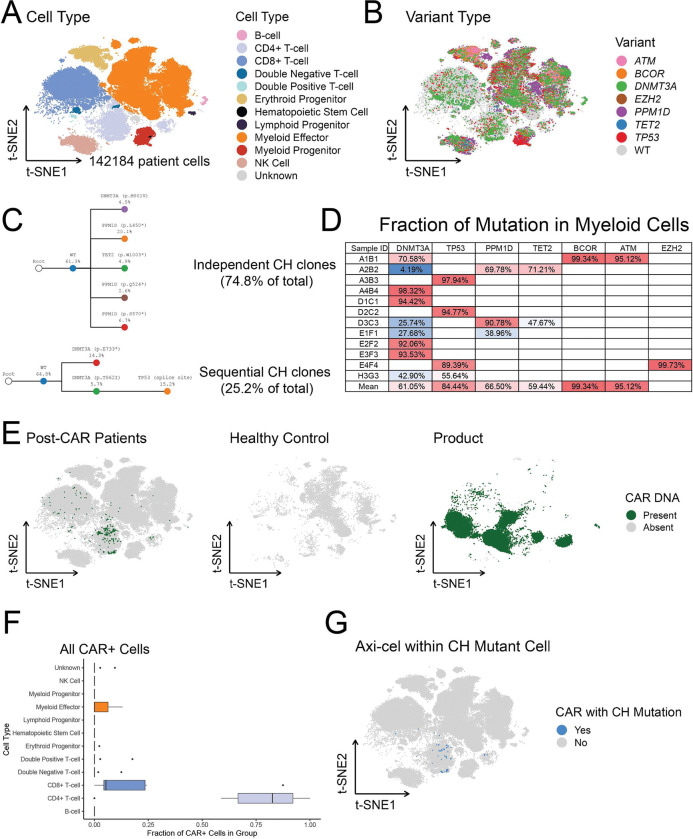
Single cell DNA analysis of post-CAR CH mutations reveals independent clonal evolution and rare CAR+ CH clones. **A)** Cell types identified via cell surface protein profiling demonstrate distinct hematopoietic compartments, including myeloid, progenitor, NK cell, and T-cell lineages. **B)** Variant distribution among cell lineages favors exclusion of DDR genes in the T-cell lineage. **C)** Clonal architecture analysis demonstrates that most post-CAR CH mutations occur as mutually exclusive clones (top panel). Sequential mutations in a single clone typically exist as an age-related CH mutation followed by a DDR CH mutation (bottom panel). **D)** Most CH clones in post-CAR bone marrow samples are myeloid-predominant. *DNMT3A* mutations exhibit the highest proportion of lymphoid involvement among CH genes, suggesting differential lineage contribution by gene. **E)** Axi-cel detection by scDNA sequencing demonstrates rare persistence in post-infusion patient samples and dominant expression in product samples. **F)** Persistent CAR populations are typically CD4+ T cells consistent with scRNA results. **G)** CAR+ cells containing CH mutations are detected in 5/12 patients though are a minority of the total CAR+ cell population and are rare relative to the T-cell population. CH, clonal hematopoiesis; CAR, chimeric antigen receptor; DDR, DNA damage response; t-SNE, t-distributed stochastic neighbor embedding.

Our prior data demonstrated the presence of post-CAR CH clones with clonal expansion. CH arises from common progenitors and is detectable across multiple hematopoietic lineages, including both the myeloid and lymphoid compartment^71,72^. To better define the clonal origin of post-infusion CH we next performed single cell DNA and antibody sequencing (DAb-seq) of n = 12 viably preserved post-CAR marrows in 11 unique patients with known CH mutations from our pre-existing panel coupled to two product specimens and one healthy control (n = 192310 total cells and 142184 post-CAR marrow cells, Figure 6A-B, **Figure S8A**, **File S8**). Amplicons were derived both from a previously validated CH panel^73^ as well as manually generated and directed against both known CH mutations and the axi-cel vector^53^. VAF of known CH mutations detected in clinical bone marrow relative to scDNA sequencing demonstrated high concordance (**Figure S8B**, R = 0.91, p<1E-5). Cells were visualized in Omiq using t-distributed stochastic neighbor embedding (t-SNE) and manually gated according to a pre-determined gating algorithm (**Figure S8C**). Simplified cell annotations were applied for downstream analyses (Figure 6A), and normalized protein expression closely corresponded to gated cell lineages (**Figure S8D**).

Each set of gene mutations per cell were defined as an independent clone and 29 total clones were identified (**Table S6**). We found that 22/29 (74.8%) unique CH containing clones contained a single mutation rather than multiple mutations existing within the same clone and possibly accounting for the previously observed clonal competition in the longitudinal tracings (Figure 6C, top panel). When multiple mutations existed together, we found that the mutational hierarchy typically involved presence of an age-related CH mutation followed by a DDR mutation indicating that chemotherapy-induced DDR mutations may confer additional fitness to pre-existing age-related CH clones (Figure 6C, bottom panel and **Table S7**). The other pattern of serial mutations observed were sequential *TP53* mutations which led to tMNs in the two MDS cases.

Comparing age related genes (*DNMT3A* and *TET2*) versus DDR genes (all others) we found that DDR genes had greater penetrance in the myeloid compartment (Figure 6D). Of all 29 clones 7/15 *DNMT3A* containing clones demonstrated a plurality of mutated cells in the lymphoid lineage. When focusing on the lymphoid lineage we found significantly increased penetrance of DDR genes in the natural killer (NK) cell type relative to other lymphoid cells (**Figure S9A**). NK cells maintain bone marrow development throughout life while T-cell proliferation shifts to the post-thymic circulation in adults. As such this mutational pattern suggests that although DDR mutations preferentially localize to myeloid cells, the progenitor mutation still exists in hematopoietic stem cells upstream of the common lymphoid and myeloid progenitor and circulating T-cells proliferating outside of the bone marrow are spared from DDR mutations acquired during chemotherapy.

### Axi-cel persists as CD4+ T-cell populations

Axi-cel was identified in 75% of distant post-infusion marrows at low levels (median 0.17% of cells) consistent with our scRNA data (Figure 6E, **Figure S9B**)^53^. Axi-cel was detected at higher levels in the two product controls (60.38% and 100% of cells, Figure 6E) and was not detected in one healthy control. In total 94.8% of axi-cel positive cells were defined as T-cells and the majority (79.8%) of axi-cel positive cells were CD4+ T-cells (Figure 6F). This was significantly different from the 28.7% of CAR− T-cells were CD4+ (p < 1E-15, Fisher’s exact test). These findings corroborate our scRNA-seq results, confirming that axi-cel predominantly persists as CD4+ T-cells in the bone marrow (Figure 2G). This directly contrasts axi-cel during initial expansion where the majority of cells are CD8+^42^.

CAR integration into the genome is not considered a primary mechanism of tMN pathogenesis. Consistent with this expectation, throughout the study axi-cel was not detected in MDS/AML samples at meaningful levels in the myeloid compartment in scRNA (**Figure S3C**), and scDNA (Figure 6F), nor did CAPP-seq analysis of MDS/AML samples support the presence of CAR-positive malignant clones (**Figure S7E**).

Based on prior reports of CH mutations contributing to either CAR T-cell fitness^48,74^ or post-CAR TCL^9,45,53^, we next assessed for the presence of CAR within clones carrying CH mutations. CAR was detected in five CH clones across five unique patients, though only three clones contained more than one CAR-positive cell (Figure 6G). All CAR integration into CH involved *TET2* and *DNMT3A*. In each case the total CAR+ CH clones detected was enriched relative to the total number expected (range 1.4 – 18x). In one clone CAR+CH+ cells made up 49/59 (83.1%) CAR+ cells providing evidence of selection (**Figure S9B**).

In the CAR+CH+ 61/63 cells were CD4+ indicating greater CD4 skew relative to the general CAR+ population (96.8% CD4+, p < 0.01, Fisher’s exact test), but most of these cells were from a single individual. In the non-CAR T-cell population (n = 47,244 cells) there was also a shift of CH+ T-cells towards the CD4+ compartment (CD4:CD8 ratio 0.665 for CH+ cells vs 0.391 for CH− T-cells, OR 1.2 and p < 1E-16 in a random effects model accounting for patient identification). This finding suggests that CAR integration into CH may facilitate CAR T-cell persistence in some cases. Further, CH-positive native T-cells have a skew towards the CD4+ compartment. Finally, though the total number of these cells was low, the detection of CAR+ CH in 5 out of 11 patients suggests that integration into CH clones may be more common than previously appreciated.

### Patient level CH expansion over time in myeloid and CAR compartments

To further validate our findings, we analyzed five PBL samples from two patients: one for CAR expansion within CH clones and another longitudinally analyzed for TP53 mutant competition and LOH (**Figure S9C-F**). Patient 102 exhibited persistent CAR integrated into a clone also containing a DNMT3A L547H mutation. Analysis of day −27 and day 7 PBMC samples revealed preferential expansion of the CAR vector within the CH clones harboring the previously described DNMT3A L547H mutation. The originator clone at day −27 made up 5.7% of T-cells expanding to 9.6% of CAR+ cells on day 7 and then 83.1% of CAR+ cells on day 1561 (**Figure S9C**). In contrast, a TET2 H1676Qfs*14 variant which was present principally in the myeloid cell line and present in only 0.46% of day −27 T-cells, was entirely excluded from the expanding CAR T cell population (**Figure S9E**). These findings illustrate that while CAR vector integration into CH clones can drive preferential expansion, integration may also be excluded from certain CH clones due to their lineage restriction.

Patient 275 received CAR19 therapy achieving CR but then died with persistent cytopenias prior to extensive workup. Near the time of death, circulating tumor DNA (ctDNA) was undetectable by cfDNA analysis; however, expansion of the TP53 R280G variant was observed in both plasma and PBLs. Sequencing of plasma and PBLs revealed clonal competition among multiple low VAF TP53 variants present pre-infusion, followed by a LOH event and subsequent expansion of the TP53 R280G clone (**Figure S3D**, 3^rd^ panel). Consistent with these findings, scDNA profiling of PBMCs at days 13, 78, and 202 showed that *TP53* variants were initially below the limit of detection at day 13, followed by expansion of multiple clones at day 78, consistent with clonal competition (**Figure S9D**, **Figure S9F**). As observed in other cases, Patient 275’s *TP53* variants were clonally exclusive except for a double-hit TP53 C176R/TP53 Y236N (**Figure S9F**). At day 202, the TP53 R280G clone exhibited LOH and underwent substantial clonal expansion within the myeloid compartment, consistent with undiagnosed tMN as the likely cause of death (**Figure S9D**). These data underscore the potential for low VAF clones to expand through clonal competition. However, the ultimate expansion after LOH of the TP53 R280G clone, rather than the double hit TP53 C176R/TP53 Y236N, indicates these interactions are complex and likely difficult to distinguish. More sophisticated modeling approaches may be required to accurately predict post-CAR tMN emergence in patients with multiple competing CH clones.

## Discussion:

Post-CAR infection and tMN are the most frequent causes of non-relapse mortality among CAR treated patients^16^. A detailed understanding of these complications is essential to ensure the safe administration of CAR T cells to current patient populations with lymphoid malignancy as well as future patient populations including those with autoimmune disease^12,44,53,63^. This study comprehensively examines the intersection of post-CAR cytopenias, tMN, and CH, establishing a mechanistic framework to understand these phenomena.

First, we show that post-CAR cytopenias, infection, and non-relapse mortality is increased in a propensity-matched cohort of CAR-treated vs HCT-treated patients. Persistent cytopenias after CAR T-cell therapy is well documented^20,21,28^ leading to morbidity and mortality. Though it is clear that CAR19 is superior to HCT in treatment of LBCL in the second line^7,75^, our results demonstrate that CAR treated responders do have reduced immune recovery and greater numbers of infections in the post-infusion setting than HCT treated controls. These findings were likely exacerbated by pandemic-era COVID exposure. Additionally, our HCT population had excellent outcomes possibly superior to previously reported rates of NRM in LBCL^76^.

To understand the pathogenesis of prolonged cytopenias, we profiled post-CAR cytopenic bone marrows, including those from patients with tMN. CAR T cells persisted in post-CAR marrow at the protein, RNA, and DNA levels up to four years after infusion validating prior reports of long-lived CAR T-cell populations^24^. These persisting CAR19 cells were predominantly CD4+ and were enriched in marrow compared to blood. B-cell progenitors were also detectable in marrow but did not mature into the periphery suggesting ongoing targeting by residual CAR T-cells.

This correlative analysis suggests that persistent CAR19 populations mediate on-target but off-tumor antigen targeting of progenitor B-cells in the marrow with subsequent consumption of B-cells, likely at the time of CD19 expression during lineage differentiation. Coupled to this persistent B-cell targeting, post-CAR bone marrow contains IFN-mediated inflammatory *non*-CAR T-cells with a paucity of growth factors and anti-inflammatory mediators in the marrow plasma. Taken together these findings support an inflamed post-CAR marrow environment with a persistently active CD4+ CAR population possibly contributing to that inflammation. The overall cause of post-CAR cytopenias is likely multifactorial, involving pre-existing hematopoietic injury from prior therapies, persistent B-cell aplasia, circulating CAR T cells, and sustained marrow inflammation following infusion (**modeled in Figure S10A**).

The reason CAR+ cells persist as CD4+ T-cell populations is unclear. In both scRNA and scDNA data these CAR+CD4+ populations are enriched relative to the CD8 dominant native T-cell population indicating lineage specificity of persistent CAR (Figure 2G, Figure 6E). CD4+ CAR persistence is consistent with prior reports of durable CD4+CAR+ populations^24^. Also, like prior reports our discovered persistent CAR+ population consists primarily of memory-like CD4+ T-cells consistent with a durable memory response driven by the chimeric receptor. This persistence may reflect the more durable memory-like signature of CD4+CAR+ cells^77^. There are known kinetic differences in CD4+ and CD8+ T-cell expansion wherein persistent antigen stimulation such as from ongoing CD19 production in the bone marrow may be more favorable to CD4+ Tcell durability relative to CD8+ T-cells which have rapid initial proliferation that is less antigen dependent^78^. The increased need for co-stimulation in CD4+ T-cells relative to CD8+ T-cells would be bypassed in CAR+ cell models where co-stimulation is engineered into the chimeric antigen receptor^79^.

Clinical molecular profiling revealed CH mutations in ~90% of evaluable patients with post-CAR cytopenias or tMN. DDR gene mutations selected for by prior chemotherapy was likely a major driver of CH in this population and may be less prevalent in patients treated in earlier lines of therapy. Many of our post-CAR cytopenic patients had clinical features consistent with CCUS^80^. There is no established treatment for individuals with this condition^81^ though the presence of CH does increase the risk for adverse outcomes and progression to hematologic malignancy in some circumstances^32,80,82^.

Myeloid malignancy is a common cause of NRM in the post-CAR setting^16^. In total 17 post-CAR patients in the study had tMN (MDS and AML). When comparing to 29 patients with tMN after HCT in an unmatched cohort, we observed no differences in molecular risk categories or clinical outcomes between post-HCT and post-CAR tMN. These findings suggest post HCT and post-CAR tMN have similar disease histories and phenotypes and do not support the notion that tMN after CAR represents a unique entity. Future multi-institutional studies incorporating histology-matched and time-matched comparisons are needed to clarify whether differences exist in the timing or clinical outcomes of post-CAR versus post-HCT tMN.

Our group of 17 post-CAR tMN predominantly arose from *TP53* mutated cell populations^83^ including frequent multi-hit loss of *TP53*^84^. The majority of post-CAR tMN occurred in patients who had received multiple cell therapies implicating cumulative marrow injury as a driver of post-CAR tMN (modeled in **Figure S10B**). CH prior to infusion did not readily predict tMN, as many cases arose from low VAF clones undetectable by standard clinical methods. Development of tMN generally required additional mutation at the alternate *TP53* allele to drive malignant transformation. The pattern of DDR mutational damage leading to subsequent clonal expansion primarily of *TP53* gene mutations matches tMN development patterns noted after HCT in multiple myeloma^34^ again supporting the similarity of post-CAR and post-HCT tMN. CH screening prior to CAR therapy as a means of risk stratification may be reasonable as more data accumulates. Importantly, such data could better inform future surveillance strategies for CH monitoring, as well as the role of key interventions to avert associated infectious complications, cytopenias, and secondary hematologic malignancies.

Using ultra-deep sequencing we detected CH in more patients than prior studies by capturing mutations with VAFs below 2%. CH mutations at <2% VAF were common and associated with delayed hematologic recovery and development of tMN. Although our CH gene panel was curated (17 pre-determined CH mutations, and inclusive only of functional variants), these findings are relevant ongoing debate over what VAF thresholds define clinically meaningful CH. Depending on methodology, some reports describe CH as ubiquitous in healthy subjects^85^, and the relevance of low VAF mutations, particularly in conditions such as CCUS, are frequently questioned^86^. Our results suggest that in the context of CAR therapy, selected low VAF mutations are biologically relevant.

To better understand the link between CH, cytopenias, and tMN, we profiled longitudinal samples. CH-associated genes expanded in post-CAR non-progressors. This finding suggests acute and persistent toxicity in the form of LD chemotherapy and direct inflammatory damage during CAR treatment likely selects for mutated progenitor populations with greater tolerance to the toxic marrow environments^87^. The observed rate of CH expansion after CAR was five to ten-fold higher than in prior reports of baseline CH expansion in healthy populations^76^ with median doubling times less than one year for many CH genes.

When querying post-CAR CH mutations at the single-cell level, we found that most mutations localized to the myeloid lineage, with *DNMT3A* mutations uniquely present in lymphoid populations^71,72^. This finding helps explain the apparent protection from tMN associated with *DNMT3A* in this and other studies^80,88^. Many mutations occurred in independent clones explaining the clonal competition noted during expansion. These clone-specific expansions are consistent with prior reports describing preferential expansion of an individual clone at the expense of other clonal populations^89^. Clones were typically independent and competed during expansion. Multi-hit clones followed a typical sequence of age-associated CH followed by DDR mutations.

We also detected CAR integration into lymphoid CH clones, particularly *DNMT3A*, in 5 of 12 patients. This finding implicates CH mutations as contributing to CAR persistence consistent with other reports^9,24,49,90^. These CAR+CH+ clonal populations may be a precursor to CAR+ TCL or may facilitate CAR persistence over time. However, our current findings indicate that CAR integration into T-cell clones is likely common, and none of the studied patients developed CAR+TCL. As such additional mutation is likely necessary to transition to CAR+ TCL and such transitions are extremely rare. *DNMT3A* mutations can promote memory-like persistence of CAR T-cells^74^ providing a mechanism for CAR persistence in CH and consistent with the persistent CD4+CAR+ memory T-cells noted in this study. Future work should aim to quantify the frequency at which CAR inserts into CH clones and whether this drives CAR persistence more broadly. This effect may be more pronounced in lentiviral 41BB vectors where post-infusion CAR+ TCLs presumably integrated into pre-existing CH have been discovered^9,43,45^.

In sum, we present a comprehensive clinical and molecular framework linking CAR persistence, inflammation, CH expansion, and tMN development. Our findings suggest that CH clones expand in an inflammatory marrow environment post-CAR, impairing hematopoiesis and sometimes progressing to malignancy. Understanding this inflammatory-CH axis may inform strategies to prevent or treat post-CAR toxicities and improve patient outcomes. Proliferation of CH clones is strongly associated with inflammatory microenvironments^91^. CH expansion particularly in *TP53* mutated clones may subsequently contribute to the development of secondary hematologic malignancy. Mechanistic understanding of these toxicities may inform methods to abrogate this persistent inflammation and the inflammatory-CH cycle that likely drives both tMN development and the ineffective hematopoiesis that underlies infectious susceptibility.

## Supplementary Material

Supplementary Files

This is a list of supplementary files associated with this preprint. Click to download.

• SupplementV11submit.pdf

## Data Availability

Sequencing data generated for this publication will be deposited in the NCBI sequence read archive database. Additional data and code used to produce figures for the manuscript is available upon request with underlying clinical data subject to IRB approval and material transfer agreement.

## Clinical Data Availability:

Demographic clinical data is included in the supplementary appendix. Additional clinical data is available upon request with an appropriate IRB approval and material transfer agreement.
